# Development and external validation of a radiomics combined with clinical nomogram for preoperative prediction prognosis of resectable pancreatic ductal adenocarcinoma patients

**DOI:** 10.3389/fonc.2022.1037672

**Published:** 2022-11-28

**Authors:** Fangqing Wang, Yuxuan Zhao, Jianwei Xu, Sai Shao, Dexin Yu

**Affiliations:** ^1^ Departments of Radiology, Qilu Hospital of Shandong University, Jinan, China; ^2^ Department of Pancreatic Surgery, Qilu Hospital of Shandong University, Jinan, China; ^3^ Shandong Provincial Hospital, Shandong University, Jinan, China

**Keywords:** contrast enhanced computed tomography, pancreatic ductal adenocarcinoma, nomogram, overall survival, preoperative prediction

## Abstract

**Purpose:**

To develop and externally validate a prognosis nomogram based on contrast-enhanced computed tomography (CECT) combined clinical for preoperative prognosis prediction of patients with pancreatic ductal adenocarcinoma (PDAC).

**Methods:**

184 patients from Center A with histopathologically confirmed PDAC who underwent CECT were included and allocated to training cohort (n=111) and internal validation cohort (n=28). The radiomic score (Rad - score) for predicting overall survival (OS) was constructed by using the least absolute shrinkage and selection operator (LASSO). Univariate and multivariable Cox regression analysis was used to construct clinic-pathologic features. Finally, a radiomics nomogram incorporating the Rad - score and clinical features was established. External validation was performed using Center B dataset (n = 45). The validation of nomogram was evaluated by calibration curve, Harrell’s concordance index (C-index) and decision curve analysis (DCA). The Kaplan-Meier (K-M) method was used for OS analysis.

**Results:**

Univariate and multivariate analysis indicated that Rad – score, preoperative CA 19-9 and postoperative American Joint Committee on Cancer (AJCC) TNM stage were significant prognostic factors. The nomogram based on Rad - score and preoperative CA19-9 was found to exhibit excellent prediction ability: in the training cohort, C-index was superior to that of the preoperative CA19-9 (0.713 vs 0.616, *P*< 0.001) and AJCC TNM stage (0.713 vs 0.614, *P*< 0.001); the C-index was also had good performance in the validation cohort compared with CA19-9 (internal validation cohort: 0.694 vs 0.555, *P*< 0.001; external validation cohort: 0.684 vs 0.607, *P*< 0.001) and AJCC TNM stage (internal validation cohort: 0.694 vs 0.563, *P*< 0.001; external validation cohort: 0.684 vs 0.596, *P*< 0.001). The calibration plot and DCA showed excellent predictive accuracy in the validation cohort.

**Conclusion:**

We established a well-designed nomogram to accurately predict OS of PDAC preoperatively. The nomogram showed a satisfactory prediction effect and was worthy of further evaluation in the future.

## Introduction

Pancreatic ductal adenocarcinoma (PDAC) is one of the most aggressive malignancies of the digestive system (1). Despite improvements in diagnosis, surgical techniques, and comprehensive treatment with follow-up, PDAC remains an intractable disease with a 5-year survival rate of 3%–15% (2). Currently, complete resection (R0) is the only potentially curative treatment for PDAC (3). However, owing to the heterogeneity of the tumor, patients with R0 resection have different outcomes (4). Thus, preoperative prediction of the accurate prognosis of PDAC patients is important to guide early individualized treatment.

For PDAC, the Tumor-node-metastasis (TNM) staging system originated from the clinical staging system of the American Joint Commission on Cancer (AJCC) classification has been widely used to guide surgical intervention and postoperative prognosis. However, accurate cancer stage can only be confirmed by a postoperative histopathologic examination and large variation in outcome occurs even in patients with the same disease stage. Therefore, there is still an urgent need for a preoperative, non-invasive and accurate method for prediction prognosis of resectable PDAC patients.

With the rapid development of radiomics, preoperative, noninvasive, accurate and high-throughput feature extraction and further analysis for medical images becomes available (5). Compared with traditional imaging studies, radiomics not only focuses on the anatomical characteristics of tumors, but also considers the complex biological behavior of tumors (6). Previous studies have showed that there is a link between radiomics and the biological behavior of a variety of malignant diseases (7). Cen C et al. suggested that computed tomography (CT) derived PDAC radiomics features can be used to Preoperatively Predict Cancer Stage (8). Eilaghi et al. suggested that CT derived PDAC texture features were correlated with overall survival and disease-free survival in patients undergoing resection (9). Although these results are very encouraging, radiological studies of pancreatic cancer prognostic risk remain limited, possibly due to limited data in current studies. In addition, the current research is mostly limited to single axial image for 2D segmentation, ignoring the large amount of information contained in the image. It has been proved that 3D segmentation can obtain more abundant tumor information than 2D segmentation (10).

Therefore, in this study, we aimed to develop and validate a nomogram that integrated preoperative CA19-9 with the radiomics score (Rad - score) of contrast enhanced computed tomography (CECT) and compared with AJCC TNM stage to accurately predict the individual survival after surgery in PDAC patients and provide a reference for clinical practice.

## Materials and methods

### Patient data

**Figure 1:**
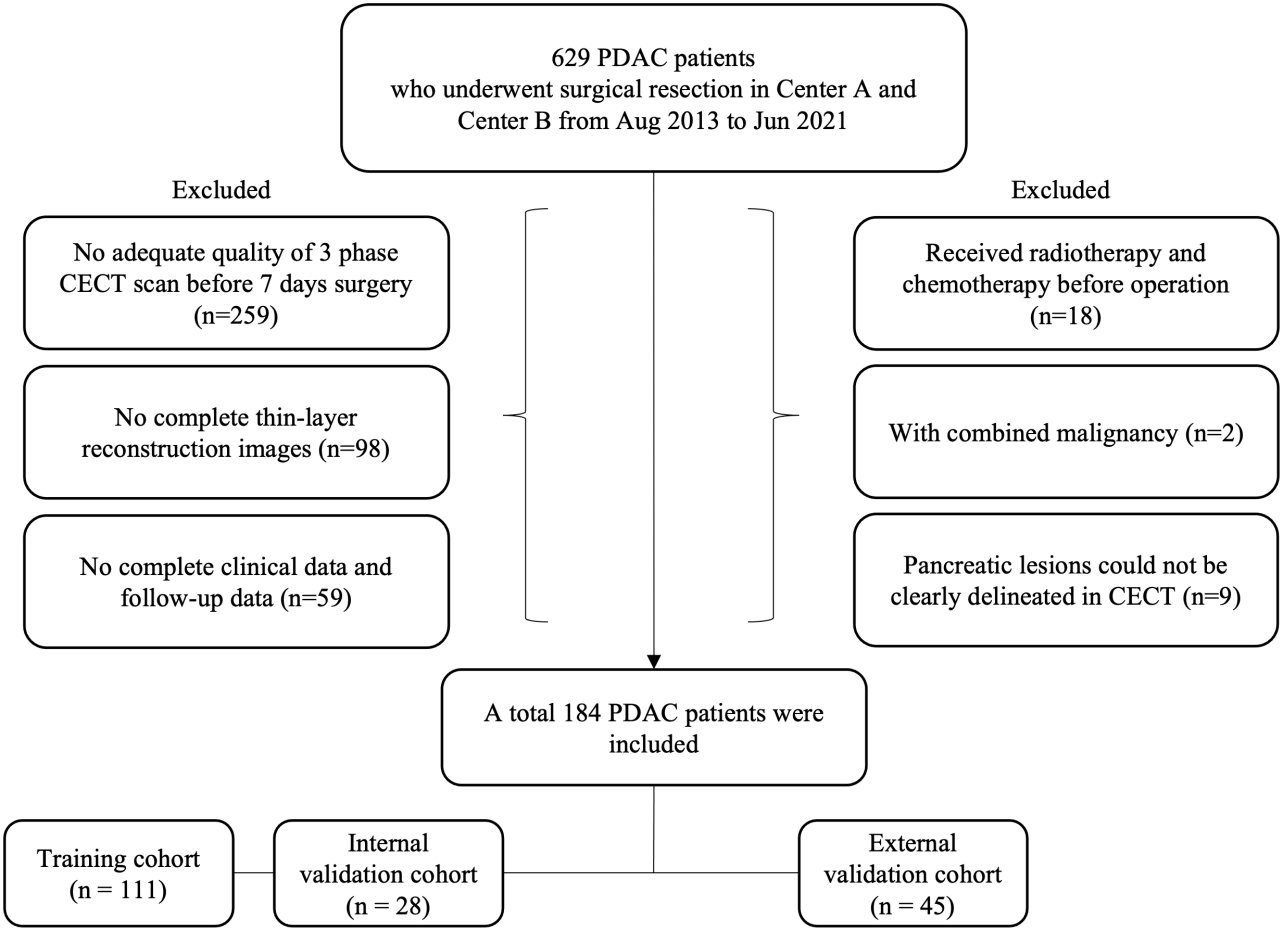
Flow chart visualizing the patient selection process.

This retrospective study was approved by the institutional review board, and the requirement for written informed consent was waived. We retrospectively reviewed the medical records of 629 patients who had undergone radical resection in Shandong University Qilu Hospital (Center A) and Shandong Provincial Hospital Affiliated to Shandong First Medical University (Center B) from Aug 2013 to Jun 2021. The inclusion criteria were as follows (1): surgically resected tumors confirmed as PDAC by postoperative pathology; (2) adequate quality of 3 phase CECT scan before surgery; (3) complete thin-layer reconstruction images (4) the availability of laboratory examinations; (5) follow-up for more than three months after surgery. We further excluded (1) patients with history of malignancies of other origin, (2) history of preoperative radiotherapy and chemotherapy, and (3) images that could not be clearly outlined on CECT. Finally, 184 patients with PDAC were screened out for analysis. Among them, 139 patients were identified in Center A as the training cohort and internal validation cohort, and 45 patients identified in Center B as the external validation cohort. The case screening criteria are shown in **Figure 1**.

### Clinical data and follow‐up

The clinical parameters including age, gender, tumor location, tumor morphology, parenchymal atrophy, dilatation of pancreatic duct, preoperative carbohydrate antigen 19-9 (CA19-9), CA125, carcinoembryonic antigen (CEA), nerve invasion and vascular invasion and pathological T, N and M stages according to AJCC TNM 8th edition (11) were extracted from the electronic medical records system. The OS of patients was obtained through clinical follow-up or telephone communication. OS was calculated from the date of surgery to the date of death caused by PDAC or at the last follow-up date on January 30, 2022.

### CT image acquisition

In center A, all patients were examined using a Philips Brilliance 256-slice helical CT scanner (Brilliance ICT, Philips, Netherlands) for three-phase CECT scans before surgery. In center B, all patients received preoperative CECT scans with multidetector row CT scanners (Somatom Force CT, Siemens, Germany). The scanning parameters were as follows: tube voltage 120 kV, variable tube current (160–600 mA) depending on the size of the patient, collimation 128×0.625 mm, rotation time 0.5 s, and layer thickness 1 mm. After unenhanced scanning, patients received approximately 65–75 mL of iopromide (350 mg I/mL, Bayer Medical, Berlin, Germany) through the cubital vein at 2.5–3.0 mL/s. CT scans of the arterial phase (AP), portal venous phase (PVP), and delay phase (DP) were performed at 25–30 s, 60–70 s, and 110–180 s after injection.

### Region of interest and segmentation

**Figure 2:**
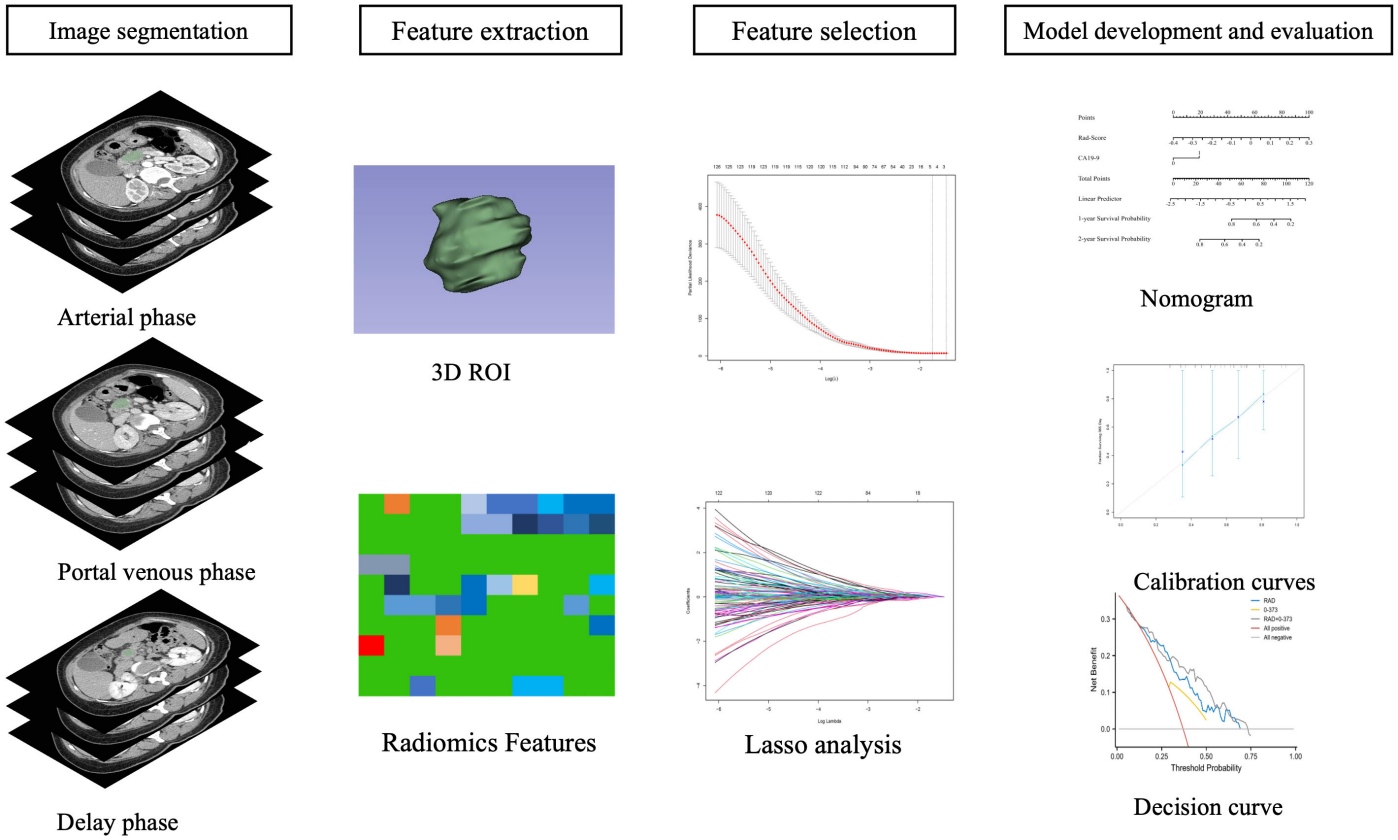
Radiomics workflow.

The radiomics workflow was shown in **Figure 2**. Before segmentation, all images were resampled to a common voxel spacing of 1 mm × 1 mm × 1 mm by using the linear interpolation algorithm to construct new data points within the range of discrete datasets of known data points to standardize spacing across all images (12). The region of interest (ROI) of the tumor was manually contoured on AP, PVP and DP CECT images using 3D-Slicer (version 4.11.2) software, avoiding calcification, vascular and cystic changes as much as possible. The two radiologists, who had 20 years and 5 years of experience in abdominal image interpretation respectively, were blinded to the clinical outcome before ROI segmentation.

### Extraction of radiomic features and radiomics score construction

1409 radiomics features were extracted from CECT images of AP, PVP and DP respectively with the open-source Python package Pyradiomics 2.2.0 (https://mics.radcloud.cn/#/login). The extracted radiomic features are calculated by gray level run length and gray level cooccurrence texture matrices, including first-order statistics, shape- and size-based features and texture features. Using the method of Z-score normalization, the extracted radiomics features were subtracted from the average value of each feature and divided by the respective standard deviation value to eliminate the restrictions imposed by each feature unit. From the training cohort, the best radiomics features were selected using the minimum absolute shrinkage and selection operator (lasso) regression, and the lasso hyperparametric regularization penalty was optimized by tenfold cross validation, optimal lambda is 0.023. Subsequently, a linear combination of coefficient weights of lasso was used to calculate the radiomics score (Rad - score) to predict the survival rate of each patient. The operator characteristic (ROC) curve was used to evaluate the predictive accuracy of Rad - score in the training and validation cohorts.

### Intra- and interobserver agreement

To assess the potential differences in tumor segmentation, the intra- and interclass correlation coefficients (ICCs) was calculated. Using the method of random stratified sampling. To evaluate the intraobserver reproducibility, the ROI delineation was performed by two radiologists (observer 1 and observer 2, WFQ with 5 years and YDX with 20 years of experience), respectively. To evaluate the intra-reader reproducibility, observer 1 repeated the ROI segmentation at a 1-month interval. ICC greater than 0.75 was considered to represent good consistency of feature extraction.

### Model development

Independent predictors of prognosis were selected using univariate multivariate Cox regression. Based on the results of the cox model, a nomogram was developed to predict 1-, 2-year OS after surgical resection, and compared with Rad-score, clinical model and AJCC TNM stage. Subsequently, the ROC curve, calibration curve and DCA curve were used to evaluate the prediction, calibration and clinical utility performance of the model.

### Statistical analysis

SPSS 23 statistical software (SPSS, Inc., Chicago, IL, USA) were used to perform the statistical analysis. Baseline data were analyzed for categorical variables and continuous variables using chi‐squared test or Mann Whitney U test, respectively. Kaplan-Meier method were used to analyze OS. For all analysis, P value< 0.05 was considered statistically significant. The “glmnet” package in R software (version 3.5.2, Vienna, Austria) was used to perform lasso-cox regression. The “pROC”, “rms” and “rmda” software packages were used to construct nomograms and calibration plots, ROC plots and DCA curves, and the “timeroc” software package was used to evaluate the accuracy of nomograms in predicting 1-year and 2-year survival rates.

## Results

### Patient characteristics

139 patients from Center A were randomly allocated to the training cohort (n = 111) and the internal validation cohort (n = 28) at a ratio of 8:2. Patients from Center B were used as an external independent validation cohort (n = 45). The median survival of the training cohort, the internal validation cohort, and the external validation cohort were 540 days, 450 days, and 460 days, respectively. The characteristics of all patients are shown in **Table 1**. Between the two centers, no significant difference in clinical characteristics (age, gender, tumor location, tumor morphology, parenchymal atrophy, dilatation of pancreatic duct, preoperative CA19-9, CA125, CEA, nerve invasion and vascular invasion and AJCC TNM Stage.

**Table 1:** Baseline parameters compared between the training, internal validation and external validation cohorts.

Characteristic	Training cohort (n = 111)	Internal validation cohort (n = 28)	External validation cohort (n = 45)	*p*
Age (years), median (IQR)	62 (55, 7)	58 (51.75, 68.3)	64 (57, 7)	0.347
Gender (%)				0.154
Male	36 (19.6%)	10 (5.4%)	22 (12%)	
Female	75 (40.8%)	18 (9.8%)	23 (12.5%)	
BMI (kg/m^2^), median (IQR)	22.77 (20.46, 25.47)	22.68 (21.67, 23.65)	23.66 (22.03, 25.71)	0.154
Diabetes, n (%)				0.861
No	82 (44.6%)	22 (12%)	33 (17.9%)	
Yes	29 (15.8%)	6 (3.3%)	12 (6.5%)	
Hypertensin, n (%)				0.543
No	79 (42.9%)	17 (9.2%)	32 (17.4%)	
Yes	32 (17.4%)	11 (6%)	13 (7.1%)	
Smoking				0.367
No	63 (34.2%)	20 (10.9%)	27 (14.7%)	
Yes	48 (26.1%)	8 (4.3%)	18 (9.8%)	
Drinking				0.189
No	58 (31.5%)	18 (9.8%)	30 (16.3%)	
Yes	53 (28.8%)	10 (5.4%)	15 (8.2%)	
Tumor location (%)				0.782
Head of pancreas	53 (28.8%)	13 (7.1%)	26 (14.1%)	
Neck of pancreas	11 (6%)	4 (2.2%)	3 (1.6%)	
Body of pancreas	33 (17.9%)	7 (3.8%)	9 (4.9%)	
Tail of pancreas	14 (7.6%)	4 (2.2%)	7 (3.8%)	
Tumor morphology, n (%)				0.444
Regular	68 (37.0%)	19 (10.3%)	24 (13.0%)	
Irregular	43 (23.4%)	9 (4.9%)	21 (11.4%)	
Parenchymal atrophy, n (%)				0.750
No	64 (34.8%)	16 (8.7%)	23 (12.5%)	
Yes	47 (25.5%)	12 (6.5%)	22 (12%)	
Dilatation of pancreatic duct, n (%)				0.425
No	20 (10.9%)	2 (1.1%)	7 (3.8%)	
Yes	91 (49.5%)	26 (14.1%)	38 (20.7%)	
CA19-9, median (IQR)	269.4 (60.7, 685.7)	424.5 (384.5, 612.0)	281.9 (76.9, 634)	0.183
CA12-5, median (IQR)	17.3 (10.7, 31.4)	20.4 (9.5, 41.6)	21.9 (12.2, 36.8)	0.409
CEA, median (IQR)	3.4 (2.1, 6.0)	3.2 (1.7, 6.0)	3.9 (2.3, 6.2)	0.739
Lymphocyte (*10^9^/L), median (IQR)	1.46 (1.18, 1.87)	1.57 (1.1, 1.77)	1.29 (1.11, 1.66)	0.725
Neutrophil (*10^9^/L), median (IQR)	3.39 (2.62, 4.4)	3.63 (2.7, 5.25)	3.47 (2.81, 4.43)	0.251
Nerve invasion, n (%)				0.684
No	32 (17.4%)	7 (3.8%)	10 (5.4%)	
Yes	79 (42.9%)	21 (11.4%)	35 (19%)	
Vascular invasion, n (%)				0.318
No	73 (39.7%)	21 (11.4%)	26 (14.1%)	
Yes	38 (20.7%)	7 (3.8%)	19 (10.3%)	
AJCC TNM Stage (IA/IB/II A/II B/III), n (%)				0.148
IA	6 (3.3%)	2 (1.1%)	4 (2.2%)	
IB	38 (20.7%)	14 (7.6%)	20 (10.9%)	
IIA	27 (14.7%)	3 (1.6%)	6 (3.3%)	
IIB	38 (20.7%)	6 (3.3%)	12 (6.5%)	
III	2 (1.1%)	3 (1.6%)	3 (1.6%)	
OS rate, % (95% CI)				
1-y	66.3% (57.7-76.3)	65.9% (49.9-86.9)	63.9% (48.1-84.8)	
2-y	38.7% (29.2-51.0)	27.3% (13.4-55.4)	37.3% (20.2-69.0)	

IQR, interquartile range; CA19-9, carbohydrate antigen 19-9; CA125, carbohydrate antigen 125 CEA, carcinoembryonic antigen; AJCC, American Joint Committee on Cancer. Bold indicates P value< 0.05 was considered statistically significant; OS, overall survival.

### Selection and modeling of radiomic features

**Figure 3:**
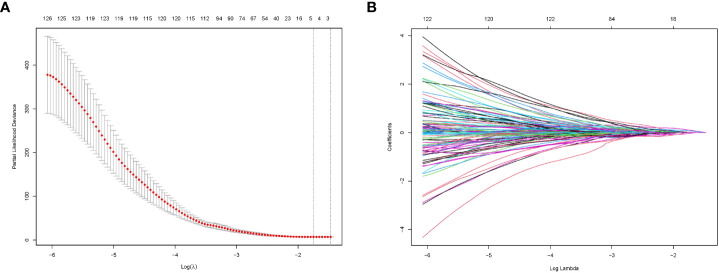
Radiomics feature selection by LASSO regression. **(A)** Selection of tuning parameters (lambda value) in the LASSO model using ten-fold cross-validation by the minimum criteria. **(B)** LASSO coefficient profiles of the radiomics features.

Research flowwork are shown in **Figure 2**. 1409 radiomics features were extracted from each patient using the python software package pyrodiomics 2.2.3 (https://mics.radcloud.cn/#/login). The intraobserver and the interobserver reproducibility showed ICCs >0.75. The lambda value with the minimum criteria in the LASSO model using 10-fold cross-validation was chosen (**Figure 3**). Finally, 4 radiomics features were confirmed (two features from DP imaging, two features from PVP imaging) and formulas for the Rad - Score were generated through a linear combination of these features weighted by the LASSO algorithm. Each feature’s coefficient was calculated from the LASSO regression method (**Table 2**).

**Table 2:** Extracted features and their coefficients.

Features	Coefficient values
original_shape_Elongation	0.1057
wavelet-LLH_glszm_GrayLevelVariance	0.0715
wavelet-HLH_glszm_ZoneEntropy	0.0066
wavelet-LHL_glszm_GrayLevelNonUniformity	-0.0352

Radiomics score (Rad - score) calculation formula = 0.1057*original_shape_Elongation+0.0715*wavelet-LLH_glszm_GrayLevelVariance+0.0066*wavelet-HLH_glszm_ZoneEntropy-0.0352*wavelet-LHL_glszm_GrayLevelNonUniformity.

Details of the Rad-score formulas are shown as follows:


Rad−Score=0.1057*original_shape_Elongation+0.0715*wavelet−LLH_glszm_GrayLevelVariance+0.0066*wavelet−HLH_glszm_ZoneEntropy−0.0352*wavelet−LHL_glszm_GrayLevelNonUniformity

### Performance evaluation of radiomic models

**Figure 4:**
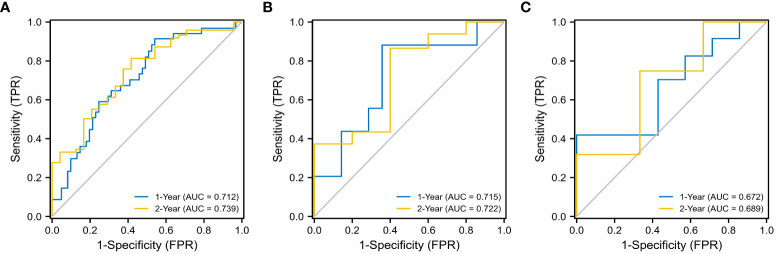
ROC analysis for 1- and 2- year OS of the radiomics models. The training cohort **(A)** (1- year: AUC = 0.712, 2 - year: AUC = 0.739), the internal validation cohort **(B)** (1- year: AUC = 0.715, 2 - year: AUC = 0.722), and the external validation cohort **(C)** (1- year: AUC = 0.672, 2 - year: AUC = 0.689).

**Figure 5:**
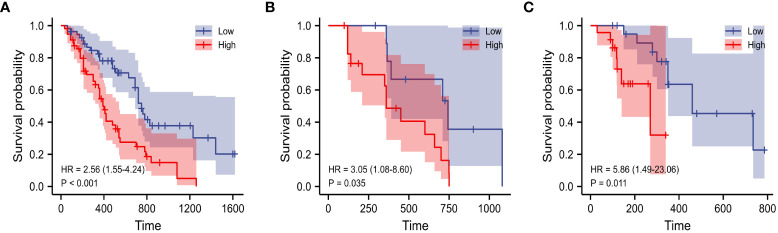
K–M analysis of Rad-score for overall survival in the training **(A)**, internal **(B)** and external validation cohorts **(C)**.

The evaluation performance of the developed Rad-score was assessed in the three cohorts using time‐dependent ROC curves. As shown in the **Figure 4**, the training cohort AUC of ROC curve predicted by radiomics for OS was 71.2% and 73.9% in 1-, 2-year, 71.5%, 72.2% in the internal validation cohort and 67.2%, 68.9% in the external validation in the same year. Next, we further explored the relationship between rad score and postoperative OS in PDAC patients. As shown in **Figure 5**, the patient survival curve was plotted using the radiomics score. In the training cohort, using the median rad score (0.002) as the cut-off point, the median OS of the high and low rad score groups in the training cohort were 750 and 400 days, respectively (*P*< 0.001), and 743 and 360 days, respectively, in the internal validation cohort (*P* = 0.035). In addition, in the external validation, the median OS of the high and low rad score groups were 460 and 270 days, respectively (*P* = 0.011).

### Univariate and multivariate COX regression analysis of the rad - score and clinical parameters

In univariate analysis, CA19-9 levels, parenchymal atrophy, AJCC TNM Stage, and Rad-Score showed significant differences in the training cohort. In multivariate analysis, Rad-score, AJCC TNM Stage, and CA19-9 level were identified as independent prognostic factors (**Table 3**).

**Table 3:** Univariate and Multivariate Cox Regression Analysis of the clinical parameters.

Characteristics	Univariate analysis		Multivariate analysis	
	Hazard ratio (95% CI)	*P* value	Hazard ratio (95% CI)	*P* value
Age (years)	0.990 (0.966-1.015)	0.435		
Gender (Male/Female)	1.265 (0.749-2.136)	0.380		
Tumor location (Head/Neck/Body/Tail)	1.125 (0.902-1.401)	0.296		
Tumor morphology (Regular/Irregular)	1.385 (0.844-2.274)	0.198		
Parenchymal atrophy (Yes/No)	0.569 (0.343-0.945)	**0.029**	0.670 (0.398-1.126)	0.130
Dilatation of pancreatic duct (Yes/No)	1.233 (0.642-2.366)	0.530		
CA19-9 (≤373/>373 U/L)	1.968 (1.200-3.229)	**0.007**	2.079 (1.253-3.450)	**0.005**
CA12-5 (≤35/>35 U/L)	0.528 (0.266-1.049)	0.068		
CEA (≤5/>5 ng/mL)	0.778 (0.448-1.351)	0.373		
Nerve invasion (Yes/No)	1.196 (0.699-2.045)	0.513		
Vascular invasion (Yes/No)	0.956 (0.580-1.576)	0.859		
Diabetes (Yes/No)	1.270 (0.735-2.195)	0.391		
Hypertension (Yes/No)	1.049 (0.616-1.788)	0.860		
Smoking (Yes/No)	0.993 (0.609-1.620)	0.979		
Drinking (Yes/No)	0.975 (0.603-1.579)	0.919		
BMI (kg/m^2^)	0.960 (0.894-1.032)	0.272		
Neutrophil (≦̸5.0/>5.0*10^9^/L)	1.136 (0.412-3.133)	0.805		
Lymphocyte (≦̸0.86/>0.86*10^9^/L)	0.587 (0.299-1.153)	0.122		
Neoadjuvant chemotherapy (Yes/No)	1.002 (0.583-1.723)	0.995		
Adjuvant chemotherapy (Yes/No)	1.328 (0.775-2.275)	0.302		
AJCC TNM Stage (IA/IB/II A/II B/III)	1.471 (1.160-1.866)	**0.001**	1.493 (1.161-1.921)	**0.002**
Rad - score	221.807 (24.285-2025.861)	**<0.001**	136.004 (14.243-1298.726)	**<0.001**

CA19-9, carbohydrate antigen 19-9; CA125, carbohydrate antigen 125 CEA, carcinoembryonic antigen; AJCC, American Joint Committee on Cancer; radiomics score, rad - score. Bold indicates P value > 0.05.

### Performance evaluation of nomogram

**Figure 6:**
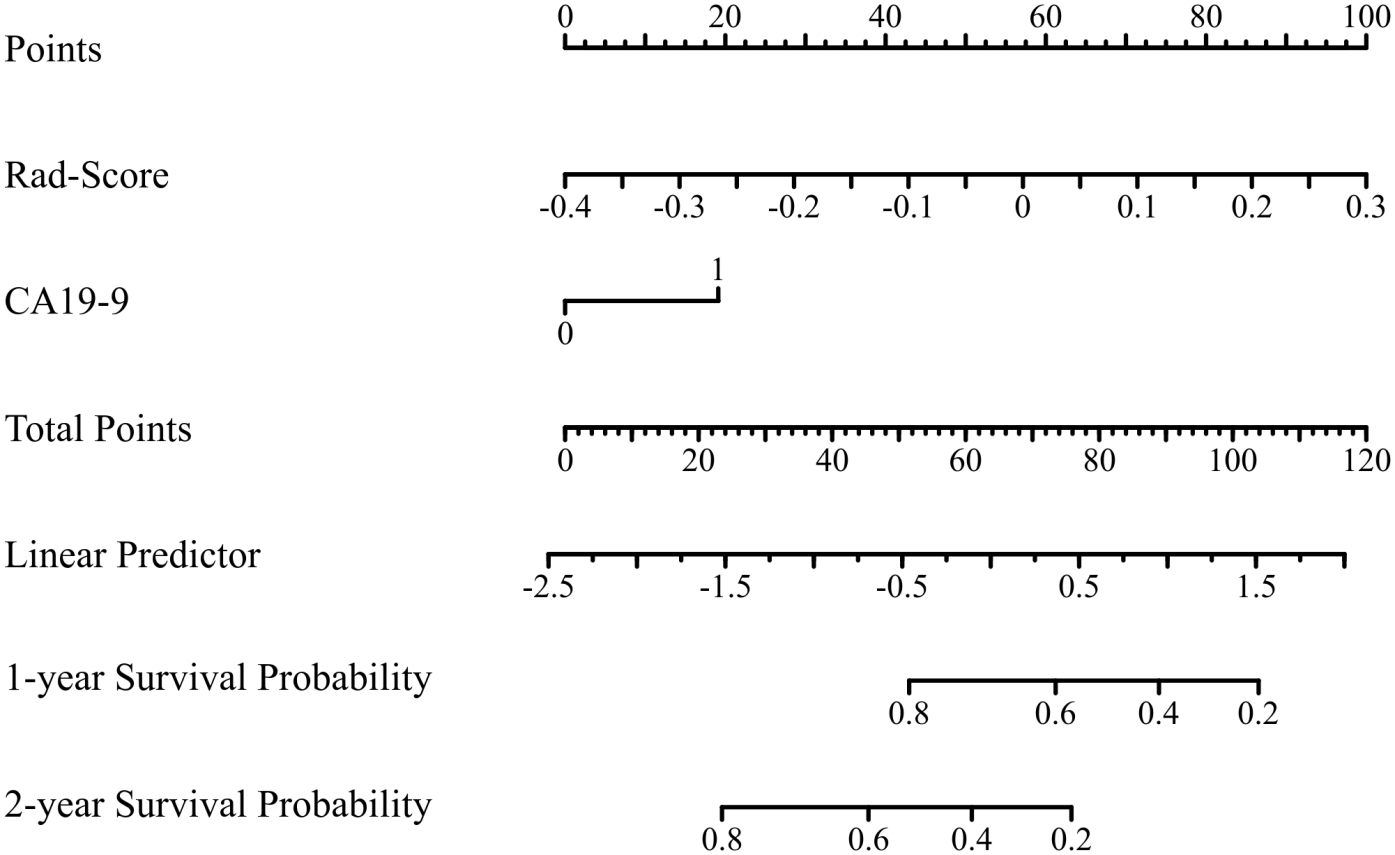
The predictive nomogram based on radiomic nomogram Rad-score and CA199 level.

Based on the result of univariate and multivariate COX analysis of the training cohort, we developed a preoperative nomogram combining CA19-9 levels and Rad-Score (**Figure 6**) and compared with AJCC TNM stage. Each factor was assigned a weighted score. The overall score for each patient was calculated and correlated with patient survival at 1-, 2-year after surgery.

**Figure 7:**
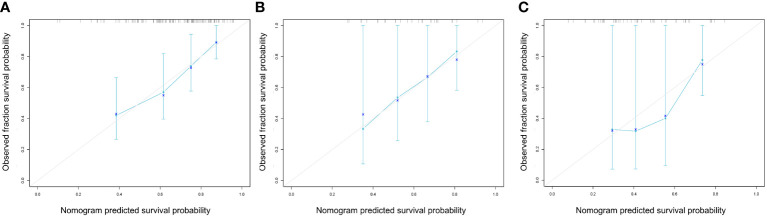
Calibration curves of the nomogram model in the training **(A)**, internal **(B)** and external **(C)** cohorts at 1 years for overall survival. These plots graph the observed (y-axis) vs predicted (x-axis) survival probabilities.

**Figure 8:**
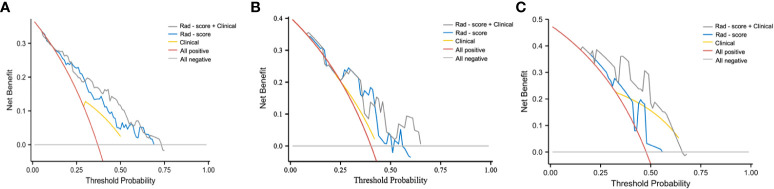
DCA curve of clinical use assessment of the radiomic signature and the radiomic nomogram in the training cohort **(A)**, internal validation cohort **(B)**, and external validation cohort **(C)**. The net benefit is shown on the y‐axis and the threshold probability is shown on the x‐axis.

As indicated by the C-index, the prediction ability of OS in the training cohort was 0.616 (95% CI:0.583-0.649) for clinical model, 0.713 (95% CI:0.680-0.746) for nomogram, and 0.614 (95% CI:0.579-0.649) for AJCC TNM stage. In the internal validation cohort, the c-index of OS was 0.555 (95% CI:0.507-0.603) for clinical model, 0.694 (95% CI:0.617-0.772) for nomogram, and 0.563 (95% CI:0.505-0.621) for AJCC TNM stage. In the external validation cohort, the c-index of OS was 0.607 (95% CI:0.534-0.681) for clinical model, 0.684 (0.619-0.748) for nomogram, and 0.596 (95% CI:0.520-0.671) for AJCC TNM stage. Compared with the clinical model or AJCC TNM stage, the nomogram showed significantly better performance (**Table 4**). The calibration curve indicated adequate consistency between estimated risks using the nomogram and the actual observed outcome in the three cohorts (**Figure 7**). The DCA showed that in three cohorts, when the threshold probability varied from 0 to 1, the nomogram achieved the most net benefit compared with a “treat all” strategy, a “treat none” strategy, and the radiomic signature (**Figure 8**).

**Table 4:** Discrimination performance of the models for overall survival.

Outcome: overall survival	C-index (95% confidence interval) Training cohort	C-index (95% confidence interval) Internal validation cohort	C-index (95% confidence interval) External validation cohort
Clinical model	0.616 (0.583-0.649)	0.555 (0.507-0.603)	0.607 (0.534-0.681)
Rad - score	0.672 (0.637-0.707) ^*†^	0.675 (0.594-0.755) ^*†^	0.639 (0.579-0.699) ^*†^
Clinical + Rad - score	0.713 (0.680-0.746) ^*†^	0.694 (0.617-0.772) ^*†^	0.684 (0.619-0.748) ^*†^
AJCC TNM Stage	0.614 (0.579-0.649)	0.563 (0.505-0.621)	0.596 (0.520-0.671)

*p value < 0.001 compared to clinical model alone; † p value < 0.001 compared to AJCC Stage.

## Discussion

In this study, we constructed and externally validated a preoperative clinical radiomics nomogram of PDAC, which integrates the three-phase CECT scanning radiomics features and preoperative CA19-9 and is significantly superior to the postoperative AJCC TNM stage in prediction OS. Taken together, the nomogram presents satisfactory predictive power for OS of PDAC, may be helpful for clinicians to identify more “invasive” tumors before operation, and select more suitable treatment methods for individuals.

We introduced serum parameters CA19-9 to develop the clinical prognostic model. CA19-9, a sialylated Lewis blood group antigen, is normally embedded on cell surfaces as gangliosides and mucins on epithelial of the pancreatic ducts and biliary tract (13). CA19-9 serum levels have a sensitivity of 79-81% and a specificity of 82-90% for the diagnosis of PDAC patients (14). It is widely considered as a useful diagnostic and prognostic biomarkers for PDAC. Yang et al. (15) reported that elevated CA19-9 was correlated with poor survival, which Hazard ratio reaching 2.648. In our study, we also confirmed that patients with higher CA19-9 levels (>373U/L) had worse OS than those with low CA19-9 levels (≤373U/L). However, CA19-9 as a marker still has limitations, including non-specific expression, false-positive results in obstructive jaundice, and so on (16). In our study, the clinical model of CA19-9 alone predicted OS with a C-index of 0.555 (95% CI:0.507-0.603) and 0.607 (95% CI:0.534-0.681) in the internal validation and external validation cohorts. Therefore, CA19-9 alone is not sufficient to accurately assess the prognosis of PDAC patients.

Since CECT has good spatial and temporal resolution with wide anatomic coverage, which is the most widely accepted technique in the diagnosis and treatment for patients with PDAC (17). However, CECT radiologic diagnosis is a subjective and qualitative preoperative diagnosis made by visual analysis. Radiomics analysis, which converts medical images into mineable high-dimensional data, is a promising method for the noninvasive assessment of tumors (18). PDAC is a tumor with low blood supply and high extracellular stromal tumor, the enhancement degree of PDAC in AP and PVP is lower than that in the DP (19, 20). Previous radiomics studies on the prognosis of PDAC mostly used only AP and PVP and used a single axial image for 2D segmentation to obtain radiomics features (21, 22). Few studies included three-phase images and used the contours of all visible tumors for 3D segmentation. However, some studies have shown that the DP, as a part of the routine CECT protocol of PDAC, has been proved to improve the diagnostic sensitivity of PDAC and thus improve the prognosis (23). Moreover, compared with 2D segmentation, 3D segmentation obtained more abundant tumor information (10).

Currently, the role of radiomics in survival estimation has been demonstrated by many previous studies in different types of cancer, including non-small cell lung cancer, breast cancer and thyroid cancer (24–26). Cassinotto et al. investigated the relationship between CT texture and DFS in resectable PDAC and found that hypo-attenuating pancreatic cancer in the portal-venous phase on CT scans showed shorter DFS (27). Sandrasegaran et al. showed that the mean value of positive pixels and kurtosis (MPP), kurtosis, entropy and skewness were significantly correlated with OS of pancreatic cancer (28). Yun et al. evaluated the relationship between DFS and CT texture features in 88 patients after radical resection of pancreatic head cancer and found that lower average values with homogeneous features (lower standard deviation and contrast and higher correlation) are significantly associated with poorer DFS (29).

In this study, 4 features were selected based on the three-phase CECT that had the greatest weights in predicting the efficacy of OS. Among them, Elongation and GrayLevelVariance came from PVP, ZoneEntropy and GrayLevelNonUniformity came from DP. On the one hand, this may be because PDAC is a tumor with insufficient blood supply, and the enhancement degree of DP is higher than that of AP and PVP (19). On the other hand, the tumorto-pancreas contrast difference was greater in the PVP than in the AP (8).In recent years, the characteristics of entropy correlation quantifies the degree of nonuniformity of image gray level and is regarded as a surrogate indicator of tumor heterogeneity (30). Our study found that entropy shows the prognostic value of OS in patients with resectable PDAC, which is consistent with the studies of Farzad et al. (22), Kim et al. (31) and Xie et al. (32). In addition, uniformity, also known as angular second-order moment, is a measure of image uniformity. Its prognostic value has also been confirmed in some studies. Cheng et al. found that uniformity was significantly correlated with DSS of advanced nasopharyngeal carcinoma (33). Low uniformity values usually appear in heterogeneous images without major intensity. Although the reduced image uniformity may reflect tumor cellularity, proliferation, hypoxia, angiogenesis and necrosis (34), the exact biological relevance of uniformity is still unclear, and it is still a challenge to link individual radiomics features with complex tumor biological processes.

The AJCC TNM staging system stratified PDAC according to three main parameters: primary tumor, regional lymph node and distant metastasis (11). This standard is still the basis for determining the prognosis of different malignancies. However, this staging system mainly focuses on anatomical and radiological features, which may oversimplify the complexity of tumor biological behavior, and the staging system relies on postoperative histopathological examination, and the results may vary greatly even in patients with the same disease stage (35). Our results explicitly clarified the difference between the prognosis estimated using our constructed nomogram and that estimated by the AJCC TNM stage, which explain the better ability of our nomogram in predicting OS than the AJCC TNM stage.

This study has several limitations. First, this study is a retrospective analysis, and there may be selection bias. A multicenter, prospective study with a larger data set will be needed in the future. Second, even if image preprocessing and feature standardization are performed, images from different centers may be affected by imaging acquisition changes. Third, Rad - score is calculated using ROI manually drawn in 3D, which is time-consuming and inconvenient in clinical work. In the future, the feasibility of automatic segmentation or semi segmentation in PDAC radiomics analysis will be required. In conclusion, we developed and externally validated a pre-operative nomogram prognostic model for PDAC. The model significantly improved prediction of OS compared to established pre-operative CA19-9 or AJCC TNM stage in PDAC. In addition, the preoperative data used in the developed risk assessment model is easy to obtain, with little additional cost, and shows good results. These advantages and results are encouraging and promising.

## Data availability statement

The raw data supporting the conclusions of this article will be made available by the authors, without undue reservation.

## Ethics statement

The studies involving human participants were reviewed and approved by Ethics Committee on Scientific Research of Shandong University Qilu Hospital. The ethics committee waived the requirement of written informed consent for participation.

## Author contributions

DY contributed to conception and design of the study. FW organized the database. YZ performed the statistical analysis. FW and YZ wrote the first draft of the manuscript. JX and SS wrote sections of the manuscript. All authors contributed to the article and approved the submitted version.

## Funding

This study was financially supported by the National Natural ScienceFoundation of China (NSFC 81771888).

## Conflict of interest

The authors declare that the research was conducted in the absence of any commercial or financial relationships that could be construed as a potential conflict of interest.

## Publisher’s note

All claims expressed in this article are solely those of the authors and do not necessarily represent those of their affiliated organizations, or those of the publisher, the editors and the reviewers. Any product that may be evaluated in this article, or claim that may be made by its manufacturer, is not guaranteed or endorsed by the publisher.
